# Benefits of Probiotics in Rheumatic Diseases

**DOI:** 10.3389/fnut.2020.00157

**Published:** 2020-09-08

**Authors:** Francesca Oliviero, Paolo Spinella

**Affiliations:** ^1^Rheumatology Unit, Department of Medicine—DIMED, University of Padova, Padua, Italy; ^2^Clinical Nutrition Unit, Department of Medicine—DIMED, University of Padova, Padua, Italy

**Keywords:** probiotics, rheumatic diseases, osteoarthritis, rheumatoid arthritis, gout, spondyloarthritis, short chain fatty acids (SCFA)

Rheumatic diseases are chronic conditions which affect a large proportion of the population worldwide (1). Among them, inflammatory chronic diseases including rheumatoid arthritis (RA), spondyloarthritis (SpA), crystal-induced arthritis (CIA), and connective tissue autoimmune diseases, such as systemic lupus erythematosus (SLE) and systemic sclerosis (SSc), are the most common. Chronic inflammation has been recognized to play a role also in osteoarthritis (OA), classically considered a non-inflammatory form of arthritis.

Although different risk factors influence their onset and development, these heterogeneous conditions share common clinical outcome including joint pain, disability, and comorbidities (2).

Over the last years increasing attention have been paid to the role of diet in the pathogenesis and progression of rheumatic diseases. It has been observed that healthy dietary habits and the consumption of foods rich in bioactive compounds, omega-3 fatty acids, and antioxidants are associated with a lower risk to develop these diseases and a less severe clinical outcome (3). In particular, an important link between gut microbiota and patients' clinical features highlighted the possibility to modulate disease progression and presentation through microbiota manipulation. In this setting, an emerging role might be attributed to probiotics, live microorganisms that, when administered in adequate amounts, confer a health benefit on the host (4).

The most commonly used are *Lactobacilli (L.), Bifidobacteria (B.)*, and *Streptococci (S.)*, either as single species or in mixed culture. Their health effects are largely recognized and supported by randomized controlled clinical trials (RCTs) and meta-analyses conducted on infectious diseases, antibiotic-associated diarrhea, irritable bowel syndrome, abdominal pain, and colitis (5, 6).

## Are There Any Evidences Supporting the Role of Probiotics in Rheumatic Diseases?

Although still limited, there's a growing interest in the possible use of probiotics in rheumatic diseases. We discuss here the evidence deriving from RCTs conducted in chronic conditions including RA, OA, gout, and SpA (Table 1). These studies have been addressed to the investigation of the effect of oral probiotic administration in ameliorating systemic inflammatory markers and disease activity indices.

**Table 1:** Randomized clinical trials carried out on the effect of probiotics in rheumatic diseases.

**References**	**Disease**	**Cases (N.)**	**Doses**	**Intervention period**	**Strains**	**Outcome measures**
Mandel et al. (7)	RA	45 (23c+22p)	1 caplet (2 billion CFU), daily	8 weeks	*B. coagulans* GBI-30, 6086	Disease activity and symptoms (ACR20 resp, HAQ-DI)
Alipour et al. (8)	RA	46 (22c+24p)	1 capsule (>0.1 billion CFU), daily	8 weeks	*L. casei* 01	Disease activity (DAS-28, hsCRP), patient's assessment (GH score, tender, and swollen joints)
Vaghef-Mehrabany et al. (9)	RA	46 (22c+24p)	1 capsule (>0.1 billion CFU), daily	8 weeks	*L. casei* 01	Pro-inflammatory (TNF-a, IL-1, IL-6, IL-12) and anti-inflammatory (IL-10) cytokines
Vaghef-Mehrabany et al. (10)	RA	46 (22c+24p)	1 capsule (>0.1 billion CFU), daily	8 weeks	*L. casei* 01	Serum patient's oxidative status
Zamani et al. (11)	RA	60 (30c+30p)	1 capsule (2 billion CFU/gr), daily	8 weeks	L. acidophilus, L. casei, B. bifidum^*^	Disease activity (DAS-28, hsCRP), serum markers of oxidative stress (NO, TAC, GSH, and MDA)
Pineda et al. (12)	RA	29 (15c+14p)	1 capsule (2 billion CFU), twice daily	12 weeks	*L. rhamnosus* GR-1, *L. reuteri* RC-14	Disease activity and symptoms (ACR20 resp, HAQ)
Lei et al. (13)	OA	461 (230c+231p)	Skinned milk (6 billion CFU), twice daily	24 weeks	*L. casei* Shirota	Clinical indices (WOMAC, VAS score, hsCRP)
Yamanaka et al. (14)	GOUT	25 (13c+12p)	Yogurt (85 million CFU), twice daily	8 weeks	*L. gasseri* PA-3	SUA levels
Kamatani et al. (15)	HYPERURICEMIA	60 (20c+20c+20p)	Yogurt (3–30 million CFU), 85 gr daily	8 weeks	*L. gasseri* PA-3	SUA levels
Jenks et al. (16)	SpA	63 (32c+31p)	1 spoon (0.1–0.4 billion CFU/gr), daily	12 weeks	*S. salivarius* K12, *B. lactis* LAFTI B94, *L. acidophilus* LAFTI L10	Patient's physical function (BASFI), disease activity (BASDAI, VAS, MASES, CRP), fatigue (MAF), bowel symptoms (DISQ), quality of life (ASQoL)
Marighela et al. (17)	SSc	73 (37c+36p)	1 sachet (1 billion CFU per prebiotic), daily	8 weeks	L. paracasei, L. rhamnosus, L. acidophillus, B. lactis^*^	Gastrointestinal symptoms, immune cell phenotype (Th1, Th2, Th17, and Treg lymphocytes)

*ACR20, American College of Rheumatology 20% improvement criteria; ASQoL, Ankylosing Spondylitis Quality of Life; BASDAI, Bath Ankylosing Spondylitis Disease Activity Index; BASFI, Bath Ankylosing Spondylitis Functional Index; CFU, colony forming units; DAS-28, disease activity score on 28 joints; DISQ, Dudley Inflammatory Bowel Symptom Questionnaire; GH, global health; GSH, total glutathione; HAQ-DI, Health assessment questionnaire disability index; hsCRP, high sensitive C-reactive protein; MDA, malondialdehyde; MAF, Multidimensional Assessment of Fatigue; MASES, Maastricht Ankylosing Spondylitis Enthesitis Score; NO, nitric oxide; OA, osteoarthritis; RA, rheumatoid arthritis; SpA, spondyloarthritis; SSc, systemic sclerosis; SUA, serum uric acid; TAC, total antioxidant capacity; Th, T helper; VAS, visual analogical scale; WOMAC, Western Ontario and Mc Master University score*.

* *Unspecified strains*.

## Rheumatoid Arthritis

Although a few RCTs have evaluated the role of probiotics in patients affected with RA, they produced promising results. Three of those studies have been conducted over a period of 8 weeks. The first study showed the effectiveness of adjunctive treatment with *Bacillus coagulans* GBI-30, 6086 lactic acid-producing bacteria in patients affected with RA (7). Although the low population size, the treatment led to a greater improvement in pain scale, patient global assessment, self-assessed disability, and serum C-reactive protein (CRP) biomarker compared with subjects randomized to placebo. The second study included 22 RA patients and 24 controls assuming placebo and demonstrated a significant decrease in DAS-28 (a composite disease activity measure in RA), serum hs-CRP, tender, and swollen joint count (8) after a daily administration of *L. casei* 01. Using the same cohort these authors observed a significant decrease in serum proinflammatory cytokines (9) but no significant effect of *L. casei* 01 supplementation in the oxidative status of the patients (10). Similar results have been obtained in a cohort of 60 RA patients (30 cases and 30 controls) (11). Besides the benefit on disease activity index, these Authors found that probiotic administration was associated with a significant improvement in insulin resistance compared with the placebo. No influence was instead demonstrated on biomarkers of oxidative stress (11).

The only trial conducted over a longer period (12 weeks) involved twenty-nine patients with RA and showed that a combination of *L. rhamnosus* GR-1 and *L. reuteri* RC-14 lead to a significant improvement in the Health Assessment Questionnaire (HAQ) score in the probiotic group but did not clinically improve RA (12).

Overall, the effect of probiotic supplementation in RA leads to a general benefit to the patients at least in the short term. This effect seems to be specie- and strain-specific.

## Osteoarthritis

Osteoarthritis represents the most frequent arthropathy among the global population and a leading cause of pain and disability (18). The processes which lead to cartilage degeneration in the OA joint have been largely described and it is now well-established that innate immunity and inflammation are involved in the pathogenesis of the disease. Nevertheless, no pharmacological treatments are available to cure or to slow down the progression of OA. This is one of the reasons which persuade patients with OA to explore complementary treatments and supplement their diet with antioxidant and bioactive natural substances (19). Clinical studies on the use of probiotics in OA are still limited but find a rationale in the recently found relationship between gut microbiota, inflammation, and OA development (20). In particular, dysbiosis has been postulated as a causal factor in the initiation and propagation of obesity-associated OA (21).

A large RCT considered the effect of *L. casei* Shirota in 461 patients with knee OA who were asked to ingest skimmed milk containing the probiotic or the placebo daily for 6 months (13). The Western Ontario and McMaster Universities Osteoarthritis Index (WOMAC), a composite score including pain and motion in OA, significantly improved in the group of patients receiving *L. casei* Shirota. These results, although limited to a single study, are encouraging and will drive further research on the benefit of probiotics in OA.

## Gout and Hyperuricemia

Gout is a frequent rheumatic disease caused by the precipitation of pro-inflammatory monosodium urate crystals in the joint. Its prevalence is rising worldwide and is associated to the substantial increase of hyperuricemia in the general population. Hyperuricemia has been shown to be associated with metabolic syndrome and to play important roles in cardiovascular and kidney diseases. Although many factors are involved in the disease, diet plays an important role in gout as it can influence circulating levels of uric acid (22) and, in particular, affect specific inflammatory pathways (23). At current, only a small RCT has evaluated the effect of probiotics in patients affected with gout (Table 1). This study showed a decrease in serum uric acid level in a subgroup of patients taking *L. gasseri* PA-3 over a period of 8 weeks (14). A reduction of uric acid has been demonstrated in a cohort of patients with hyperuricemia receiving two doses of *L. gasseri* PA-3 compared to placebo (15).

The hypouricemic effect of probiotics have been only recently evidenced in animal studies. *Lactobacillus Brevis* DM9218, for instance, have been observed to reduce serum uric acid in mice fed a high fructose diet. The mechanism is interesting as it is associated to the degradation of inosine, an intermediate metabolite of uric acid (24). Aside from the uric acid lowering effect, we think that the possible benefit of probiotics in gout might also be considered in light of their anti-inflammatory, anti-cytokine potential (as discussed below), which might target inflammasome activation and the production of IL-1β, the most important inflammatory mediators in gout.

## Spondyloarthritis

Spondyloarthritis (SpA) encompass different clinical phenotypes including psoriatic arthritis and ankylosing spondylitis. It has been suggested that dysbiosis might represent an important risk factor in these diseases (25). Patients with SpA often present symptomatic or asymptomatic signs of bowel disease and an increased intestinal permeability which allows luminal bacterial species and antigens to interact with the host immune system more readily. In our view, probiotics could find a rationale in these subjects for their capacity to strengthen the epithelial barrier and to modulate intestinal microbiota. Only an RCT study has been carried out in SpA patients so far. A probiotic combination has been orally administered in patients with active ankylosing spondylitis and the effect on clinical indexes has been investigated over a period of 12 weeks (Table 1). The group of patients taking probiotics did not demonstrate significant benefit over the placebo group (16).

A pilot non-controlled study carried out on 18 patients with SpA associated with ulcerative colitis showed that a combination of *L. acidophilus* and *L. salivarius* ameliorate SpA through a reduction in disease activity and patients' perception of pain (26). Overall, these studies did not draw any firm conclusion and data from other SpA such as psoriatic arthritis are still lacking. However, considering the possible association between dysbiosis and the development of the disease, it would be worth investigating the role of probiotics in the prevention of these diseases through large-scale longitudinal studies.

## Other Rheumatic Diseases

No clinical studies have been conducted so far on the effect of probiotics supplementation in other rheumatic diseases such as SLE and SSc. Both these autoimmune conditions have been associated with altered intestinal microbiota, but the relationship between dysbiosis and the pathogenesis of the disease is not completely clear.

Despite the lack of clinical evidences, some experimental studies are encouraging. Using mouse models of SLE some authors have demonstrated that probiotic supplementation ameliorates disease activity, Treg regulation, and typical comorbidities occurring in SLE including cardiovascular complications (27, 28). More recently, lactobacilli administration appeared to delay SLE progression via mechanisms involving Treg induction and IL-10 production (29).

As far as SSc is concerned, some RCTs have been carried out only in patients with gastrointestinal involvement. One of these has shown that an 8-week probiotics supplementation did not have any effect in reducing gastrointestinal symptoms but lead to a decrease in Th17 cell levels indicating an immunomodulatory capacity of the probiotic strains used (Table 1) (17). Another trial confirmed the inefficacy of probiotics in systemic sclerosis associated gastrointestinal disease but SSc clinical outcomes were not addressed in that study (30).

## Possible Mechanisms of Action

While the local effects of probiotics on gut health have been well documented, the mechanisms underlying their systemic anti-inflammatory and immunomodulating potential are still largely unclear.

Evidences from *in vitro* studies (31, 32) and animal models of disease (33, 34) indicate short chain fatty acids (SCFA) as possible mediators of these functions. SCFA including butyrate, acetate, and propionate, are microbiota metabolites deriving from intestinal bacteria fermentation of dietary fibers.

Besides their local effect on gut health, SCFA have shown to significantly impact on peripheral immune response and systemic inflammation through the regulation of different immune cell functions (35). Butyrate has demonstrated to suppress antigen-induced arthritis in mice by affecting both B and T cell development. For instance, it inhibits germinal center B cell and plasmablast differentiation (36) and cytokine production by invariant NKT cell, a cell subset implicated in synovial inflammation and joint destruction (33). An increase polarization toward a T regulatory (Treg) phenotype with a decrease in Th17 cell has been observed in a different animal model of RA along with the inhibition of pro-inflammatory cytokines (34).

Beyond SCFA, additional mechanisms can be proposed to explain the beneficial activity of probiotics in rheumatic diseases. Among them, the regulation of T helper and T reg cell functions and the induction of immune tolerance appears as the most significant.

*L. casei* Shirota has shown to effectively prevent the incidence of autoimmune diseases affecting the release of cytokines from antigen presenting cells and, consequently, the differentiation of lymphocytes toward effector T cell-specific subsets (37). This is of particular interest given that the Th1-Th17 response play an important role in the initial phase of the development of autoimmune disorders, including autoimmune rheumatic diseases.

Probiotic bacteria have been shown to induce a Treg immune response in experimental immune disorders including RA (38). In particular, they have been shown to favor the conversion of T cells into Tregs expessing the forkhead box transcription factor FOXP3 and potentiate the suppressor activity of naturally occurring Tregs (38).

This capacity to induce immune tolerance have been also associated to a reduced pathogenic humoral immune response in the mouse model of collagen-induced arthritis where the oral feeding of animals with *L. Casei* Shirota suppressed the abnormal antibody production evocated by type 2 collagen immunization (39).

The reduction of the antibody immune response has been also associated with the upregulation of FoxP3 positive Treg cells, an increase in anti-inflammatory, and a decrease in pro-inflammatory cytokines (40) in the same model. From the clinical point of view, this effect might have a beneficial impact on systemic inflammation and, as a consequence, play a role in the prevention of cardiovascular comorbidities associated to chronic sustained inflammation in rheumatic diseases.

The modulation of TLR signaling might represent an additional mechanism through which probiotics exert their effects in rheumatic diseases. Besides their ability in binding specific TLRs and affect downstream signaling, it has been shown that some lactobacillus strains can promote the expression of proteins which negatively regulate TLR activity, thus attenuating the inflammatory response induced by different pathogens (41).

Other possible mechanisms of actions may include the modulation of tryptophan metabolism and adenosine signaling as observed in other models (42, 43). Of interest, the activation of adenosine receptors by specific probiotic stains is implicated in the suppressive effect of Treg toward T effector cells (Th1-Th2-Th17) (43) and might reinforce their beneficial role in autoimmune diseases.

## Conclusions

Rheumatic diseases are frequent chronic inflammatory conditions often involving autoimmune processes. Most of these diseases are currently well cured especially after the development of different biological drugs which target specific proteins and receptors. Others, like OA and certain severe forms of SSc, have still no cure and represent a burden in terms of patients' quality of life.

It is well-known that the pharmacological therapy plays a fundamental role in the management of rheumatic patients and it is usually a treatment for life.

However, in the last two decades, there have been an explosion of studies indicating how lifestyle, and mainly diet, can influence chronic disease course and pathogenesis. In this context, dietary control and supplementation might be fundamental in supporting standard treatments. Concerning probiotics, the results of the RCTs discussed above are still limited to firmly conclude their effect in improving disease activity or progression, and even less in preventing disease development. However, their promising anti-inflammatory and immunomodulating properties are worth to be investigated by larger population studies, in order to identify their possible functional interest in supporting standard therapies and disease activity control in rheumatic diseases.

## Author Contributions

All authors listed have made a substantial, direct and intellectual contribution to the work, and approved it for publication.

## Conflict of Interest

The authors declare that the research was conducted in the absence of any commercial or financial relationships that could be construed as a potential conflict of interest.
